# Supplementary material for: Preparation of Fe3O4-Ag Nanocomposites with Silver Petals for SERS Application
Source: Nanomaterials (Basel). 2021 May 13;11(5):1288. doi: 10.3390/nano11051288 (PMC8153338; doi:10.3390/nano11051288)
Supplement: Supplementary file 1 [file nanomaterials-11-01288-s001.zip › nanomaterials-1177311-supplementary.pdf]

nanomaterials

MDPI

Supplementary Materials

Preparation of Fe_3O_4-Ag Nanocomposites with Silver Petals for SERS Application

Thi Thuy Nguyen [1,2,*], Fayna Mammeri [3], Souad Ammar [3], Thi Bich Ngoc Nguyen [2], Trong Nghia Nguyen [2], Thi Ha Lien Nghiem [2], Nguyen Thi Thuy [4] and Thi Anh Ho [5]

1 Graduate University of Science and Technology, Vietnam Academy of Science and Technology, 18 Hoang Quoc Viet, Cau Giay, Hanoi 10000 , Vietnam
2 Institute of Physics, Vietnam Academy of Science and Technology, 18 Hoang Quoc Viet, Cau Giay, Hanoi 10000, Vietnam; ntbngoc@iop.vast.ac.vn (T.B.N.N.); trongnghia@iop.vast.ac.vn (T.N.N.); halien@iop.vast.ac.vn (T.H.L.N.)
3 ITODYS, Université de Paris, CNRS, UMR 7086, 15 rue J-A de Baïf, 75013 Paris, France; Fayna.mammeri@univ-paris-diderot.fr (F.M.) ; ammarmer@univ-paris-diderot.fr (S.A.)
4 Institute of Materials Science, Vietnam Academy of Science and Technology, 18 Hoang Quoc Viet, Cau Giay, Hanoi 10000, Vietnam; ntthuy@ims.vast.ac.vn
5 Faculty of engineering physics and nanotechnology, VNU University of Engineering and Technology, 144 Xuan Thuy, Cau Giay, Ha Noi 10000, Vietnam; anhht2508@gmail.com
* Correspondence: thuynt@iop.vast.ac.vn; Tel.: +84-2438347953

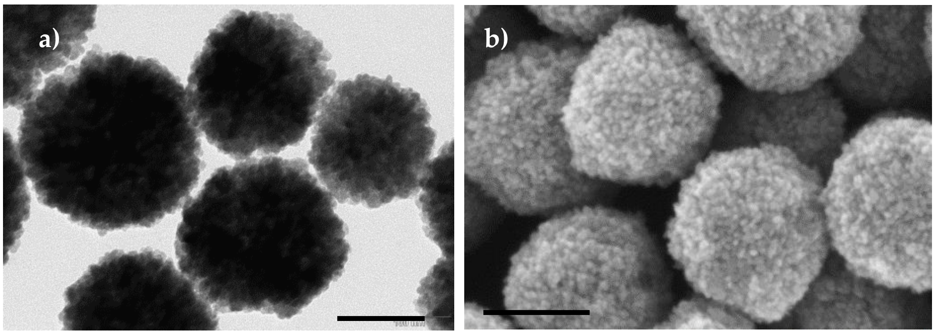

Figure S1. Representative TEM (**a**) and SEM (**b**) images of Fe_3O_4 particles. The scale bar is of 100 nm in all the micrographs.

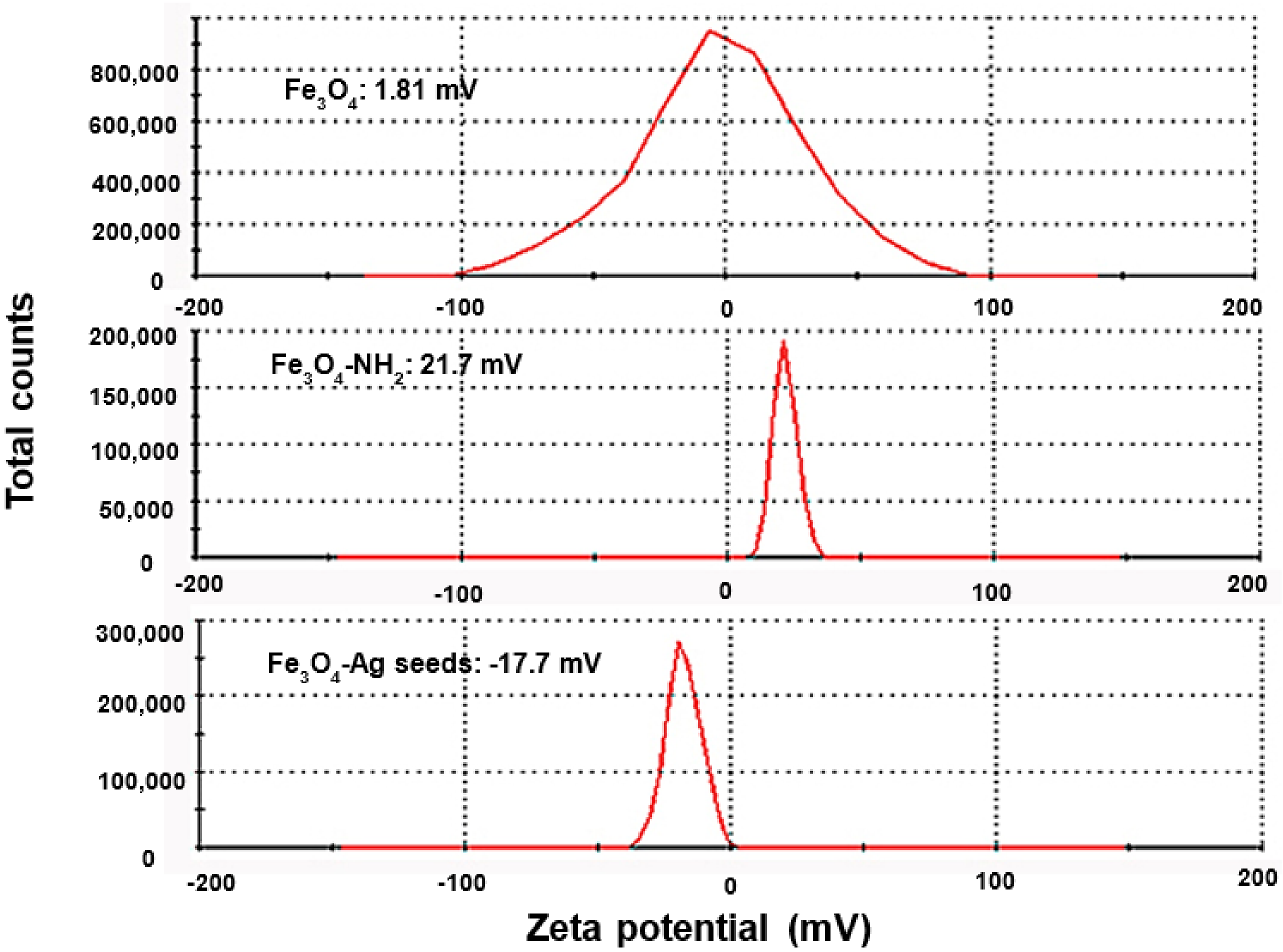

Figure S2. Zeta potential distribution of the Fe_3O_4-Ag seeds compared to that of Fe_3O_4 particles coated with APTES and when dispersed in deionized water.

Table S1. EDX elemental analysis of Fe_3O_4 particles, Fe_3O_4-Ag seeds, Fe_3O_4-Ag nanocomposites produced with PVP for a r value of 10 and 2, and without PVP for a r value of 10.

Sample	% Weight		% Atomic	
	Fe	Ag	Fe	Ag
Fe_3O_4 particles	66.9	0	37.72	0
Fe_3O_4-Ag seeds	64.14	0.24	34.86	0.07
Fe_3O_4-Ag with PVP (r=10)	8.59	76.12	38.57	0.31
Fe_3O_4-Ag with PVP (r=2)	17.83	57.92	21.98	13.07
Fe_3O_4-Ag without PVP (r=10)	0	94.76	0	73.19

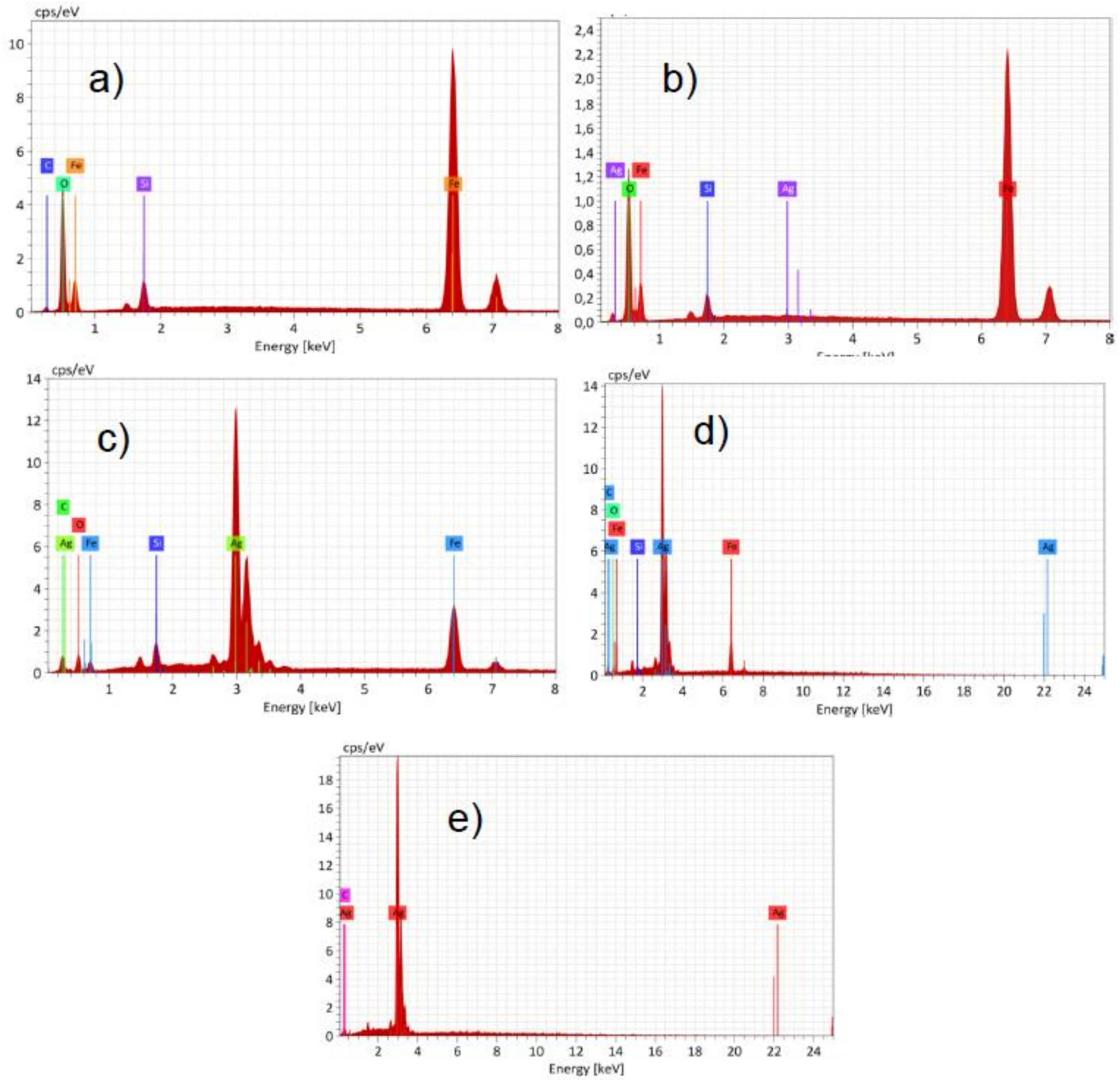

Figure S3. EDX analysis of: (**a**) Fe_3O_4 particles; (**b**) Fe_3O_4-Ag seeds; (**c–e**) Fe_3O_4-Ag nanocomposites produced with PVP for a r value of 10 and 2 and without PVP for a r value of 10, respectively.

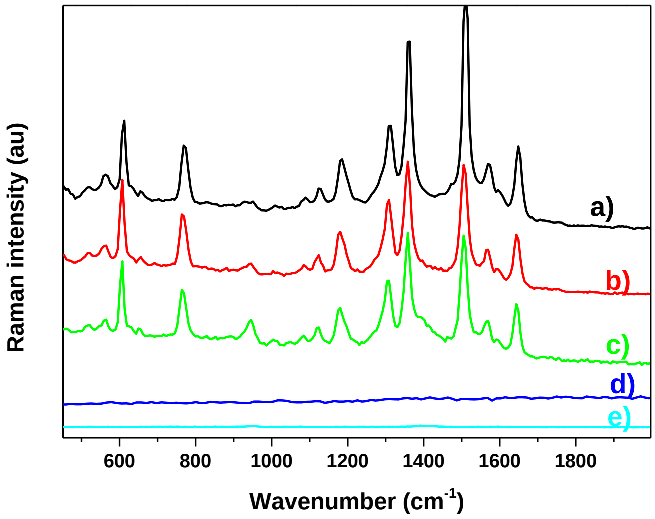

Figure S4. SERS spectra of R6G at 10^{-6} adsorbed on (**a**) Fe_3O_4-Ag nanocomposites with PVP (r = 10), (**b**) Fe_3O_4-Ag nanocomposites with PVP (r = 2), (**c**) Fe_3O_4-Ag nanocomposites without PVP (r = 10), (**d**) Fe_3O_4-Ag seeds, (**e**) Fe_3O_4 particles.

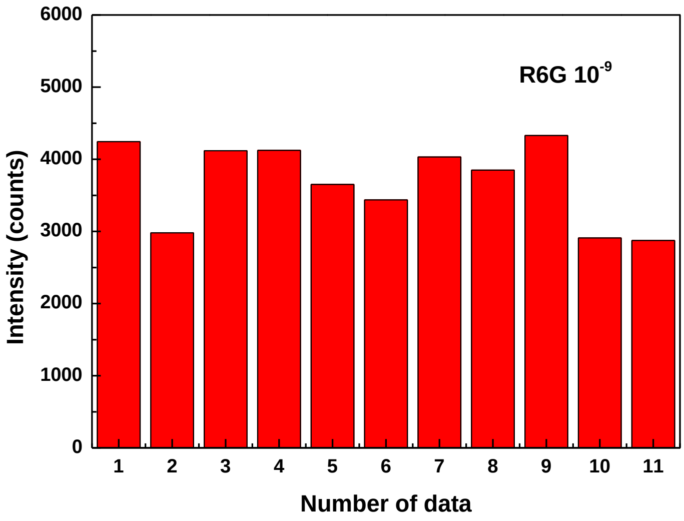

Figure S5. SERS intensity of R6G at 10^{-9} adsorbed on Fe_3O_4-Ag nanocomposites with PVP (r = 10).

Table S2. Assignment of Raman bands in spectra of R6G.

Raman Shift (cm^{-1})	Assignment
612	C-C-C ring in-plane bending
774	C-H out-of-plane bending
1185	C-H in-plane bending
1313	Aromatic C-C stretching
1363	Aromatic C-C stretching
1510	Aromatic C-C stretching
1574	Aromatic C-C stretching
1654	Aromatic C-C stretching
